# Differentially Methylated Epiloci Generated from Numerous Genotypes of Contrasting Tolerances Are Associated with Osmotic-Tolerance in Rice Seedlings

**DOI:** 10.3389/fpls.2017.00011

**Published:** 2017-01-19

**Authors:** Hui Xia, Weixia Huang, Jie Xiong, Shuaigang Yan, Tao Tao, Jiajia Li, Jinhong Wu, Lijun Luo

**Affiliations:** ^1^Shanghai Agrobiological Gene CenterShanghai, China; ^2^College of Plant Sciences and Technology, Huazhong Agricultural UniversityWuhan, China

**Keywords:** epigenetic marker, drought-resistance, methylation-sensitive amplification polymorphism, breeding, epigenotype

## Abstract

DNA methylation plays an essential role in plant responses to environmental stress. Since drought develops into a rising problem in rice cultivation, investigations on genome-wide DNA methylation in responses to drought stress and in-depth explorations of its association with drought-tolerance are required. For this study, 68 rice accessions were used for an evaluation of their osmotic-tolerance related to 20% PEG6000 simulated physiological traits. The tolerant group revealed significantly higher levels of total antioxidant capacity and higher contents of H_2_O_2_ in both normal and osmotic-stressed treatments, as well as higher survival ratios. We furthermore investigated the DNA methylation status in normal, osmotic-stressed, and re-watering treatments *via* the methylation-sensitive amplification polymorphism (MSAP). The averaged similarity between two rice accessions from tolerant and susceptible groups was approximately 50%, similar with that between two accessions within the tolerant/susceptible group. However, the proportion of overall tolerance-associated epiloci was only 5.2% of total epiloci. The drought-tolerant accessions revealed lower DNA methylation levels in the stressed condition and more de-methylation events when they encountered osmotic stress, compared to the susceptible group. During the recovery process, the drought-tolerant accessions possessed more re-methylation events. Fourteen differentially methylated epiloci (DME) were, respectively, generated in normal, osmotic-stressed, and re-watering treatments. Approximately, 35.7% DME were determined as tolerance-associated epiloci. Additionally, rice accessions with lower methylation degrees on DME in the stressed conditions had a higher survival ratio compared to these with higher methylation degrees. This result is consistent with the lower DNA methylation levels of tolerant accessions observed in the stressed treatment. Methylation degrees on a differentially methylated epilocus may further influence gene regulation in the rice seedling in response to the osmotic stress. All these results indicate that DME generated from a number of genotypes could have higher probabilityies for association with stress-tolerance, rather than DME generated from two genotypes of contrasting tolerance. The DME found in this study are suspected to be good epigenetic markers for the application in drought-tolerant rice breeding. They could also be a valuable tool to study the epigenetic differentiation in the drought-tolerance between upland and lowland rice ecotypes.

## Introduction

The term epigenetic refers to heritable variations in gene regulation, resulting from covalent modifications of DNA and its associated chromatin proteins, without changing the underlying nucleotide sequences (Shea et al., 2011; Becker and Weigel, 2012). Plant epigenetics received increasing attention as it has been reported to play important roles in plant development and adaptive responses to environmental stresses (Steimer et al., 2004; Boyko and Kovalchuk, 2008; Marie and Jerzy, 2011; Sahu et al., 2013). Cytosine methylation is a conserved epigenetic mechanism that mainly occurs at cytosine bases in all sequence contexts in plants. These include symmetric CG, CHG (in which H = A, T, or C), and asymmetric CHH contexts (Henderson and Jacobsen, 2007). Among these three cytosine contexts, CpG dinucleotides are typically clustered around the regulatory region of a gene, especially in the promoter and first exon, which can impact its transcriptional regulation (Zhao and Han, 2009; Garg et al., 2015). Moreover, the transcriptional regulation *via* DNA methylation may further more alter the phenotypic appearances of plants (Angers et al., 2010; Ou et al., 2012; Róis et al., 2013). Consequently, DNA methylation is suspected to play an important role in plant tolerances to environmental stresses (Paun et al., 2010; Dowen et al., 2012; Bräutigam et al., 2013).

Genome-wide DNA methylation under various abiotic and biotic stresses have been recorded for various plant species (Kou et al., 2011; Wang et al., 2011, 2015; Karan et al., 2012; Colaneri and Jones, 2013; Fan et al., 2013; Shan et al., 2013; Gao et al., 2014). Researchers also aspired to disclose the associations between DNA methylation and stress-tolerance *via* artificial generation of DNA methylation differences between two genotypes of contrasting tolerances (Wang et al., 2011; Gayacharan and Joel, 2013; Gao et al., 2014; Ferreira et al., 2015; Garg et al., 2015; Wang W. et al., 2016). However, DNA methylation is genotype-specific (Karan et al., 2012). Results could be therefore varied among studies that utilize different genotypes (Wang et al., 2011; Gayacharan and Joel, 2013; Zheng et al., 2013). Additionally, there were always severe epigenetic variations between two genotypes (Wang et al., 2011; Karan et al., 2012; Garg et al., 2015; Wang W. et al., 2016). It is a challenge to separate tolerance-associated epigenetic variance from neutral genotype-specific variance between a single tolerant and a single susceptible genotype (Wang W. et al., 2016).

Asia cultivated rice (*Oryza sativa*) is one of the most important cereal crops and it is very sensitive to drought stress. Drought has become a rising problem for rice production and causes severe yield loss (Luo, 2010). In addition to the underlying genetic mechanisms, the epigenetic basis (particularly DNA methylation) of drought-tolerance received an increasing amount of attentions during recent years (Wang et al., 2011; Zheng et al., 2013; Garg et al., 2015). Osmotic stress (also called hypertonic dehydration) always occurs simultaneously with the drought and its tolerance is a vital part of drought-resistance (Mahajan and Tuteja, 2005; Farooq et al., 2009). Compared to the complexity of drought-resistance in the field, the osmotic tolerance can easily be evaluated in well-controlled laboratory conditions by cultivating rice in various hypertonic solutions. Based on these considerations, we focused our study on rice tolerance to osmotic stress and its associations with DNA methylation.

The methylation-sensitive amplification polymorphism (MSAP) technique is modified from the amplified fragment length polymorphism (AFLP) by using a pair of isoschizomeric restriction enzymes with different sensitivities to site-specific cytosine methylation. This is a powerful and economic method to explore genome-wide DNA methylation in plant responses to biotic and abiotic stresses with a considerable number of samples (Paun et al., 2010; Schulz et al., 2013). We thus applied the MSAP technique to investigate DNA methylation in rice seedlings under normal conditions, mild osmotic conditions (simulated by 20% PEG6000), and re-watering conditions subsequent to drought. Furthermore, a number of osmotic-tolerant and osmotic-susceptible accessions were involved in this study to explore the associations between DNA methylation and tolerance to osmotic stress in rice.

## Materials and methods

### Plant materials

Sixty-eight rice accessions of the *japonica* subspecies were involved in this study. Their osmotic-tolerance related physiological traits and DNA methylation were investigated under normal condition, osmotic-stressed condition, and re-watering condition. The rice accession S1 (pre-evaluated survival ratio of 6.8%) was used as a susceptible reference, while the rice accession S41 (pre-evaluated survival ratio of 15.3%) was used as a tolerant reference.

### Experimental treatments and sampling strategies

Seedlings of each rice accession were cultivated in normal nutrient solution (Table S1) for 5 days after germination on a 96-well plate. They were placed in a growth chamber (14 h of daytime at 30°C and 10 h of night at 20°C with 70% relative humidity). Each rice accession had three plates (Plate#1, Plate#2, and Plate#3) with 24 individuals per plate and anther plate (Plate#4) with 48 individuals (Figure S1). After 20 days of growing in the normal nutrient solution, seedlings in Plate#2, Plate#3, and Plate#4 were treated with 20% PEG6000 to simulate osmotic stress, while seedlings in Plate#1 were kept in normal solution as control (CK). Given the large amount of rice accessions, three biological replicates were harvested from Plate#1 and Plate#2 (defined as OS) to measure the physiological traits 24 h after osmotic treatment as they began to show signs of slight leaf rolling. Each biological replicate contained three individual seedlings mixed harvested. Three biological replicates were always applied in measurements of physiological traits under stresses in rice (Huang et al., 2009; Du et al., 2010; Li et al., 2016). Moreover, three further seedlings were mixed harvested to extract their DNA/RNA. Plate#3 was re-watered with normal nutrient solution 2 days after osmotic treatment and then cultivated for another 2 days when most of these seedlings were recovered from osmotic stress. Three rice seedlings in Plate#3 (defined as RO) were mixed sampled for DNA extraction to investigate their MSAP epigenotypes. Plate#4 was re-watered 5 days after the osmotic treatment (severe osmotic stress) and the survival ratio of seedlings was calculated for each rice accession to estimate the osmotic-tolerance.

### Procedures of DNA extraction and MSAP genotyping

Total genomic DNA was extracted following the common cetyltrimethyl ammonium bromide (CTAB) protocol. Three seedlings per material were mixed to include the epigenetic variation within a rice accession. The procedure of MSAP was described in detail in our previous study (Zheng et al., 2013). Fifteen selective primer combinations were used for this study (Table S2). The 5′ end of the selective primer was labeled with fluorescent dyes. The PCR products were analyzed on an ABI 3130XL (Applied Biosystems, USA) using ROX500 as internal standard. The resulting chromatograms were analyzed and scored by Peakscanner ver. 1.0. This method enabled accurate separation and scoring of MSAP bands (Xia et al., 2016). The repeatability of MSAP genotyping was quantified via the scoring error, which was counted based on three biological replicates of 36 randomly selected rice accessions.

Comparisons of the banding patterns of EcoRI/HpaII and EcoRI/MspI reactions resulted in four conditions of a particular fragment, representing different types of DNA methylation (Table 1). The “0/0” type could be determined as hyper-methylation if it was altered to other epigenotypes in one of the three experimental conditions (CK, OS, and RO). However, it was considered as the uninformative genetic mutant when it scored “0/0” in all three conditions. To be more cautious, an epilocus with more than 10% uninformative epigenotypes would be excluded from our data set (Online Supplementary Data Sheet 1). Epiloci of lower epigenetic diversity (frequency of the minor epigenotype below 5%) under all three treatments were also excluded from further analyses.

**Table 1:** **The methylation type, status, and methylation degree for each MSAP epigenotype**.

**Epigenotype (H/M)**	**Methylation type**	**Methylation status**	**Methylation degree**
1/1	Non-methylation	CCGG	0
		GGCC	
0/1	Full-methylation	C^me^CGG or C^me^CGG	2
		GG^me^CC GGCC	
1/0	Hemi-methylation	^me^CCGG	1
		GGCC	
0/0	Hyper-methylation	^me^CCGG or ^me^C^me^CGG	3
		GGC^me^C GG^me^C^*me*^C	
0/0	Genetic mutation	CC***T***G	NA
		GG***A***C	

### Measurements and comparisons of physical traits of tolerant and susceptible materials in normal and osmotic treatments

Five osmotic-tolerance related physiological traits were assessed in CK and OS treatments, including relative water content (RWC), total soluble protein (TSP), total anti oxidant capacity (AOC), H_2_O_2_ content, and the content of malonaldehyde (MDA). The RWC was estimated as: (fresh weight-dry weight)/(saturate weight-dry weight). The measurements of TSP (Product# A045-2), AOC (Product# A015), H_2_O_2_ content (Product# A064), and the content of MDA (Product# A003-2) were assessed following protocols of their corresponding test kits (Table S3) from the Nanjing JianCheng Bioengineering Institute, China. Further information on the utilized kits can be found on the website of the Nanjing JianCheng Bioengineering Institute (http://elder.njjcbio.com/html_en/search.php) accessible via their respective codes (A003, A015, A045, and A064).

### Data analysis

#### Comparisons of physical traits between normal and osmotic treatments and between tolerant and susceptible materials

The tolerance to osmotic stress was quantified via the survival ratio of rice seedlings counted in Plate#4. The measured physiological traits were compared either between normal and osmotic treatments via paired *t*-test or between tolerant and susceptible genotypes via independent *t*-test. A correlation analysis was conducted between the measured physiological traits with the survival ratio using the method of Pearson's coefficient, in which *p*-values were corrected by FDR (false discovery rate). All these analyses were conducted with the software SPSS ver. 15.0.

#### Similarity in DNA methylation, methylation levels, and alterations of epigenotypes in responses to stress and recovery between tolerant and susceptible groups

The similarity in DNA methylation between two rice accessions was defined as the proportion of an epilocus possessing the same epigenotype to the total epiloci. We then calculated the averaged similarity within each group (tolerant or susceptible) or between two groups for CK, OS, and RO treatments. The DNA methylation level of an epilocus was calculated as the proportion of (Type II + Type III + Type IV)/(Type I + Type II + Type III + Type IV). The proportion of de-methylation (from higher methylation degree to lower methylation degree), re-methylation (from lower methylation degree to higher methylation degree), and unchanged events were calculated for each epilocus. Five alteration patterns of DNA methylation were recorded from CK to OS to RO and their proportions were calculated (Table S4). The statistical differences (*p* < 0.05) of DNA methylation level and the proportion of de-methylation/ re-methylation/ unchanged events between tolerant and susceptible groups were analyzed via independent *t*-test. Correlation analyses of DNA methylation levels as well as the proportion of each alteration pattern were also conducted with the survival ratio via Pearson's coefficient. All these *p*-values were FDR corrected.

#### Determination of the differentially methylated epilocus between tolerant and susceptible materials and their associations with osmotic tolerance

If a MSAP locus was associated with tolerance to osmotic-stress, different epigenotypes on this epilocus should impact the survival ratio. We thus defined the tolerance-associated epilocus if rice accessions conferring different epigenotypes at an epilocus represent significantly different survival ratios. This was either determined via independent *t*-test (two epigenotypes) or one-way ANOVA using the SNK method (more than two epigenotypes). The hierarchical analysis of molecular variance (AMOVA) implemented in GenAlex ver. 6.43 was conducted between osmotic-tolerant and osmotic-susceptible groups (Excoffier et al., 1992; Peakall and Smouse, 2012). The ΦCT calculated by the hierarchical analysis of molecular variance (AMOVA) based on the epigenetic variation could represent the level of epigenetic differences between osmotic-tolerant and osmotic-susceptible materials. Thus, epiloci of the top 5% highest ΦCTs between groups of different tolerances were determined as differentially methylated epiloci (DME) between both groups. The principal coordinates analysis (PCoA) was conducted via GenAlex ver. 6.43 using the covariance-standardized method to test whether the osmotic-tolerant and osmotic-susceptible accessions could be separated by these differentially methylated epiloci, as well as by the total epiloci. This PCoA analysis was based on the tri distance matrix calculated from the data of differentially methylated epiloci or total epiloci via GenAlex ver. 6.43. We hypothesized that these differentially methylated epiloci should significantly influence the osmotic tolerance. To test this, we calculated the proportion of tolerance-associated epiloci in differentially methylated epiloci, as well as their proportion in total epiloci.

#### Gene annotation at candidate epiloci and their expressions in normal and osmotic conditions

PCR products between 100 and 400 bp of the MSAP primer combination E14-HM36 were recycled from 1.5% agorose gel. They were purified and sent to sequencing *via* Illumina HiSeq 2500 (Biomarker Technologies Co., LTD., Beijing, China) with 10 M bases for each MSAP primer combination. The quality-controlled reads were mapped to the reference genome MSU 7.0. Although large amount of sequenced DNA fragments were present in the PCR products (Figure S2), only the fragments of higher abundance could have resulted from the selective amplification and form the detectable signal in the chromatogram. Therefore, only the DNA fragments with depth >100 (4.2% of total fragments) were considered to be potential epiloci detected in this study. We calculated the molecular length (bp)of the sequenced fragment (depth > 100) between the cutting sites of both restriction enzymes (*EcoRI*: G|AATTC; *HpaII*/*MspI*: C|CGG). If the length of a fragment matched the molecular weight of ascored MSAP band (±0.5 bp), it was then determined as the corresponding scored epiloci on the chromatogram (Table S5). If a differentially methylated epilocus also registered as a tolerance-associated epilocus and had a unique location mapped to the rice genome, it was selected as a candidate epilocus and its impact on gene expression was tested.

Expressions of two genes (LOC_Os03g51020 and LOC_Os07g16224) at epiloci EL265 and EL268 were consequently quantified via qPCR in at least five tolerant and susceptible materials in the CK and OS treatments (Table S6). Total RNA was extracted with the TRNzol-A+ Total RNA Reagent (TIANGEN, Beijing, China). cDNA was culled from total RNA with the PrimeScript® RT reagent Kit (Takara Biotechnology, Dalian, China) according to the manufacturer's instructions. Oligoprimers are listed in Table S5. Real-time PCR was performed using Hard-Shell® 96-Well PCR Plates (BIO-RAD, USA) with the CFX96TM Real-Time System (BIO-RAD, USA). Each reaction contained 10 μl of 2x SYBR Premix Ex TaqTM (Takara Biotechnology, Dalian, China), 20 ngcDNA, and 0.1 μM gene-specific primers in a final volume of 20 μl. The thermal cycle used was 95°C for 30 s, followed by 40 cycles at 95°C for 5 s, and 60°C for 31 s, with an additional dissociation stage. Each material was subjected to three individual biological replicates and three technique replicates for qPCR. *Actin* was used as reference to calculate the relative expression levels of these genes. A web analysis tool “Rice Functional Related gene Expression Network Database (Rice FREND)” (http://ricefrend.dna.affrc.go.jp/) was used to analysis the co-expression network of the DME-associated gene and GO enrichment (FDR<0.05) was also conducted for its top 50 co-expressing gene to explore its relevant biological functions (Sato et al., 2013).

## Results

### Survival ratio and physiological responses of rice seedlings under osmotic stress

The survival ratios of the rice seedlings ranged from 0.0 to 50.1% during severe osmotic stress induced via 20% PEG6000 treatment that lasted for 5 days. Since the survival ratio of the tolerant reference accession was 10.2%, materials with survival ratio above 10.0% were determined as tolerant materials (Figure S3, Table S7). Rice accessions with survival ratios below 10.0% were then determined as susceptible accessions, although the survival ratio of the susceptible reference was recorded as 0.

**Figure 1:**
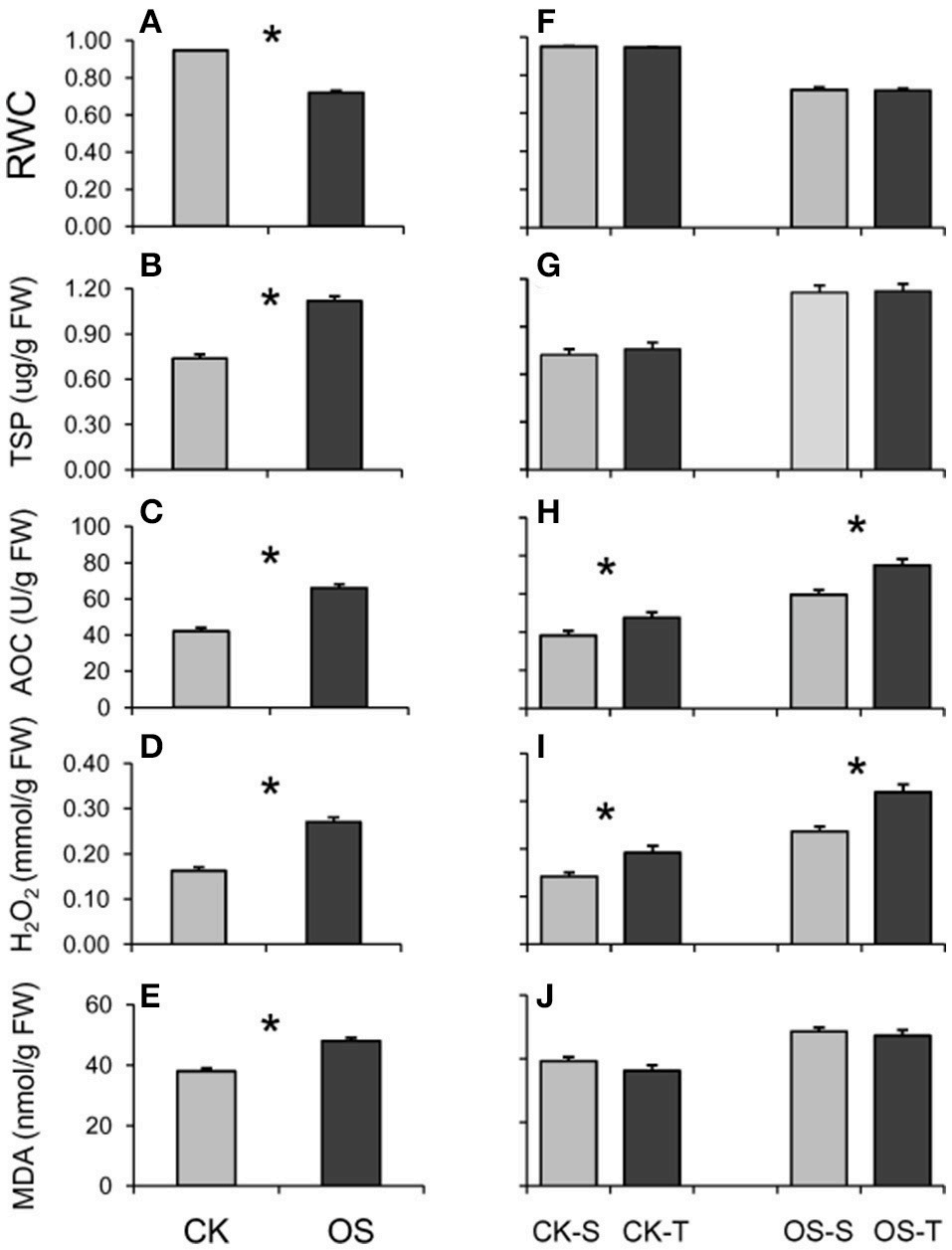
**Relative water content (RWC) (A,F)**, total soluble protein (TSP) **(B,G)**, total antioxidant capacity (AOC) **(C,H)**, content of H_2_O_2_ **(D,I)**, and content of malonaldehyde (MDA) **(E,J)** measured between normal (CK) and stressed (OS) conditions **(A–E)** or between tolerant and susceptible groups **(F–J)**. The asterisk indicates significance at *p* < 0.05. Bars indicate standard errors.

Generally, all measured physiological traits were greatly and significantly altered when treated with a 20% PEG6000 solution for 8 h (Figure 1). E.g., the level of RWC significantly decreased (Figure 1A), while the levels of TSP, AOC, H_2_O_2_, and MDA significantly increased (Figures 1B–E). Compared to the susceptible group, the tolerant group had significantly higher levels of AOC and H_2_O_2_ in both control and osmotic conditions (Figures 1H, I), suggesting that AOC and H_2_O_2_ both played essential roles in osmotic stress tolerance of rice. This result was further confirmed with a correlation analysis in which the levels of AOC and H_2_O_2_ in both CK and OS conditions significantly and positively correlated with the survival ratio (Table 2).

**Table 2:** **Correlations between measured traits by the method of Pearson's coefficient**.

**Trait**	**Survival**	**AOC-CK**	**AOC-OS**	**H_2_O_2_-CK**	**H_2_O_2_-OS**	**MDA-CK**	**MDA-OS**	**TSP-CK**	**TSP-OS**	**RWC-CK**	**RWC-OS**
Survival	–	0.397	0.450	0.524	0.474	−0.196	−0.149	0.116	0.085	−0.123	−0.029
AOC-CK	**0.005**	–	0.639	0.853	0.380	−0.310	−0.497	0.536	−0.250	−0.052	0.115
AOC-OS	**0.000**	**0.000**	–	0.660	0.601	−0.354	−0.301	0.165	−0.152	−0.270	−0.145
H_2_O_2_-CK	**0.000**	**0.000**	**0.000**	–	0.670	−0.196	−0.377	0.316	−0.168	−0.181	0.097
H_2_O_2_-OS	**0.000**	**0.005**	**0.000**	**0.000**	–	−0.044	−0.085	−0.098	−0.007	−0.262	−0.068
MDA-CK	0.269	**0.037**	**0.012**	0.269	0.953	–	0.566	0.004	−0.046	0.233	−0.178
MDA-OS	0.426	**0.000**	**0.045**	**0.009**	0.738	0.000	–	−0.069	0.214	0.080	−0.388
TSP-CK	0.579	**0.000**	0.384	**0.035**	0.677	1.000	0.814	–	0.018	0.270	−0.158
TSP-OS	0.738	0.112	0.419	0.376	1.000	0.953	0.211	1.000	–	0.018	−0.162
RWC-CK	0.552	0.930	0.085	0.330	0.093	0.151	0.755	0.085	1.000	–	0.145
RWC-OS	1.000	0.579	0.426	0.677	0.814	0.330	**0.005**	0.404	0.384	0.426	–

*The values in the upper are Pearson's coefficients while the values in the below diagonal matrix are false discovery rate (FDR) corrected p–values. FDR < 0.05 are in bold*.

### DNA methylation of rice accessions under CK, OS, and RO treatments

**Figure 2:**
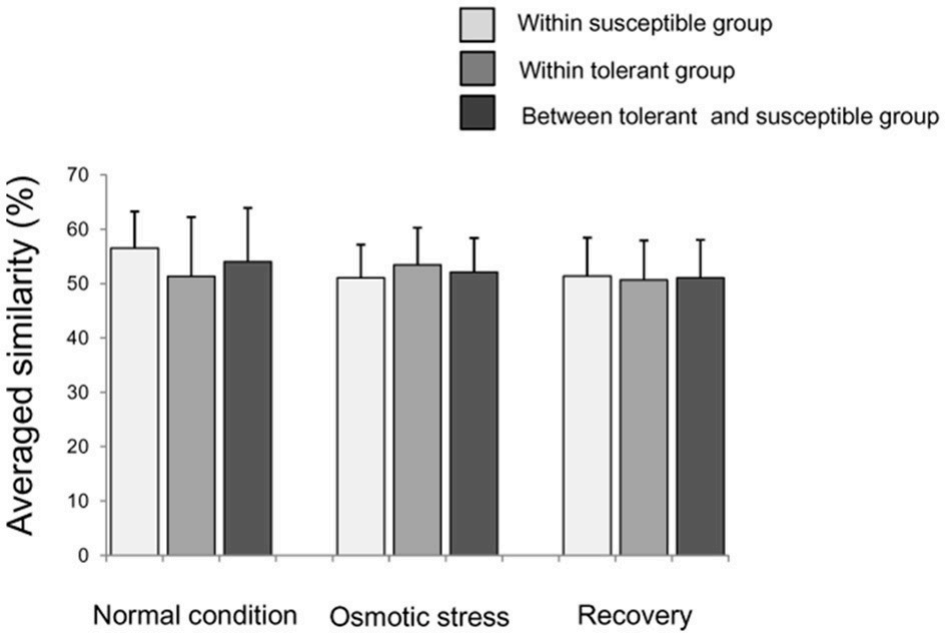
**Averaged similarity of DNA methylation between two rice accessions within the tolerant/susceptible group or between two groups**. Bars indicate standard deviations. The similarity is calculated as the proportion of epiloci with same epigenotypes to the total epiloci between two rice accessions.

A total of 293 informative epiloci scored on these 15 MSAP primers with an average scoring error of 7.40% (Table S2). The proportions of the uninformative genotype and/or missing data were 3.13, 3.27, and 3.40%, respectively, for CK, OS, and RO conditions (Online Supplementary Data Sheet 1). The average similarity of DNA methylation between a tolerant accession and a susceptible accession were 54.0 ± 9.9% (mean ± SD), having no difference the average similarity between two susceptible (56.6 ± 6.7%) or two tolerant accessions (51.9 ± 10.9%) in CK (Figure 2). The situations were similar in OS and RO treatments (Figure 2).

In general, the methylation levels of rice materials increased from CK to OS and from OS to RO (Table 3). The proportions of all four MSAP epigenotypes were almost identical between the tolerant and susceptible group in the CK and RO condition, as well as for the methylation levels (Table 3). However, the proportions of the four MSAP epigenotypes showed significant differences between the tolerant and the susceptible group in the OS condition (Table 3). E.g., materials of the tolerant group exhibited significantly lower methylation levels than materials in the susceptible group (Table 3).

**Table 3:** **The proportion of each epigenotype and the methylation level in the susceptible and tolerant groups under the normal (CK), stressed (OS), and recovery (RO) conditions**.

**Condition**	**Material**	**Number**	**00**	**01**	**10**	**11**	**Methylation levels**
CK	Susceptible	40	13.0 ± 0.7	23.4 ± 0.7	17.7 ± 1.0	45.8 ± 1.2	54.2 ± 1.2
	Tolerant	28	14.9 ± 1.4	23.0 ± 1.0	18.7 ± 1.3	44.1 ± 1.9	55.9 ± 1.9
	Overall	68	13.8 ± 0.7	22.9 ± 0.6	18.1 ± 0.8	45.1 ± 1.0	54.9 ± 1.0
OS	Susceptible	40	19.4 ± 0.8^*^	18.4 ± 0.8^*^	21.8 ± 0.7^*^	40.3 ± 1.0^*^	59.6 ± 1.0^*^
	Tolerant	28	16.5 ± 0.9^*^	22.1 ± 0.6^*^	16.0 ± 0.7^*^	45.6 ± 1.2^*^	54.4 ± 1.2^*^
	Overall	68	18.0 ± 0.6	22.8 ± 0.5	17.3 ± 0.6	41.8 ± 0.8	58.1 ± 0.8
RO	Susceptible	40	26.9 ± 0.9	23.5 ± 0.9	19.2 ± 0.8	30.4 ± 0.8	69.6 ± 0.8
	Tolerant	28	28.1 ± 1.6	22.8 ± 1.0	18.5 ± 1.2	30.5 ± 1.1	69.5 ± 1.1
	Overall	68	27.4 ± 0.8	23.2 ± 0.7	19.0 ± 0.7	30.4 ± 0.7	69.6 ± 0.7

*Values in bold with asterisks indicate significant differences (p < 0.05) between the susceptible and tolerant groups*.

For most occasions, the tolerant and susceptible group had similar alteration patterns of DNA methylation from CK to OS or from OS to RO. However, it is worth noting that significantly higher proportions of de-methylation events were observed in the tolerant group from CK to OS, while significantly higher proportions of de-methylation events were observed in the susceptible group from OS to RO (Table 4). The five alternation patterns of DNA methylation from CK to OS to RO were also very similar between tolerant and susceptible groups (Table S4).

**Table 4:** **Alterations of epigenotypes from the normal condition (CK) to the osmotic-stressed condition (OS) and from the osmotic-stressed condition (OS) to the recovery (OS)**.

	**From CK to OS**			**From OS to RO**		
**Methylation change**	**Susceptible**	**Tolerant**	**Overall**	**Susceptible**	**Tolerant**	**Overall**
00_01	4.22 ± 0.35^*^	5.57 ± 0.63^*^	4.78 ± 0.34	5.77 ± 0.44	4.74 ± 0.39	5.35 ± 0.31
00_10	2.82 ± 0.28	2.87 ± 0.28	2.84 ± 0.20	3.42 ± 0.23	3.05 ± 0.27	3.27 ± 0.17
00_11	1.77 ± 0.20^*^	3.16 ± 0.60^*^	2.34 ± 0.28	3.35 ± 0.22	2.67 ± 0.27	3.07 ± 0.18
01_10	2.37 ± 0.23	1.96 ± 0.21	2.20 ± 0.16	1.70 ± 0.18	2.25 ± 0.22	1.93 ± 0.14
01_11	4.21 ± 0.31	4.58 ± 0.37	4.36 ± 0.24	4.18 ± 0.34	3.84 ± 0.35	4.04 ± 0.25
10_11	5.40 ± 0.56	7.29 ± 0.83	6.18 ± 0.48	3.98 ± 0.41	3.29 ± 0.36	3.69 ± 0.28
De-methylation	20.79 ± 0.78^*^	25.44 ± 2.00^*^	22.70 ± 0.98	22.41 ± 0.80^*^	19.85 ± 0.84^*^	21.36 ± 0.60
01_00	6.04 ± 0.40	5.07 ± 0.45	5.64 ± 0.30	6.27 ± 0.44^*^	8.18 ± 0.57^*^	7.06 ± 0.37
10_01	1.99 ± 0.18	2.49 ± 0.28	2.19 ± 0.16	2.39 ± 0.25	1.84 ± 0.27	2.16 ± 0.18
10_00	4.84 ± 0.38	4.20 ± 0.32	4.58 ± 0.26	6.61 ± 0.44	5.93 ± 0.48	6.33 ± 0.33
11_00	4.18 ± 0.42	3.57 ± 0.63	3.93 ± 0.36	7.11 ± 0.51	8.24 ± 0.76	7.58 ± 0.44
11_01	4.89 ± 0.40	5.31 ± 0.48	5.07 ± 0.30	5.91 ± 0.42	6.26 ± 0.51	6.05 ± 0.32
11_10	7.60 ± 0.50	6.16 ± 0.63	7.01 ± 0.40	8.56 ± 0.52	8.69 ± 0.78	8.61 ± 0.44
Re-Methylation	29.54 ± 1.23	26.80 ± 1.55	28.41 ± 0.97	36.85 ± 1.11	39.14 ± 1.45	37.79 ± 0.89
00_00	5.78 ± 0.69	4.37 ± 0.50	5.20 ± 0.46	6.89 ± 0.49	5.76 ± 0.65	6.42 ± 0.40
11_11	28.33 ± 0.84	28.5 ± 11.14	28.41 ± 0.68	18.82 ± 0.56	20.72 ± 0.90	19.60 ± 0.51
10_10	5.23 ± 0.37	4.48 ± 0.44	4.92 ± 0.29	5.57 ± 0.33	4.60 ± 0.48	5.17 ± 0.28
01_01	10.32 ± 0.35	10.40 ± 0.59	10.35 ± 0.31	9.47 ± 0.40	9.93 ± 0.45	9.66 ± 0.30
Unchanged rate	49.67 ± 0.97	47.76 ± 1.56	48.89 ± 0.86	40.74 ± 0.77	41.01 ± 1.25	40.85 ± 0.68

*Values in bold with asterisks indicate significant differences (p < 0.05) between the susceptible and tolerant groups*.

### Correlations between parameters of DNA methylation and measured physiological traits

The survival ratio of the rice seedlings after osmotic stress treatment correlated negatively with the “methylation level in OS” and the “de-methylation ratio from OS to RO,” but correlated positively with the “re-methylation ratio from OS to RO” (Table S8). However, the correlations were weak as indicated by FDR correlated *p*-values (Table S8). The parameter of “methylation level in CK” correlated positively with AOC-CK and TSP-CK, but correlated negatively with MDA-OS (Table S8). The parameter of “re-methylation from CK to OS” correlated negatively with AOC-CK and TSP-CK, while the parameter of “de-methylation from CK to OS” correlated positively with AOC-CK (Table S8).

### Impact of differentially methylated epiloci on the survival ratio of rice seedlings under osmotic stress

**Figure 3:**
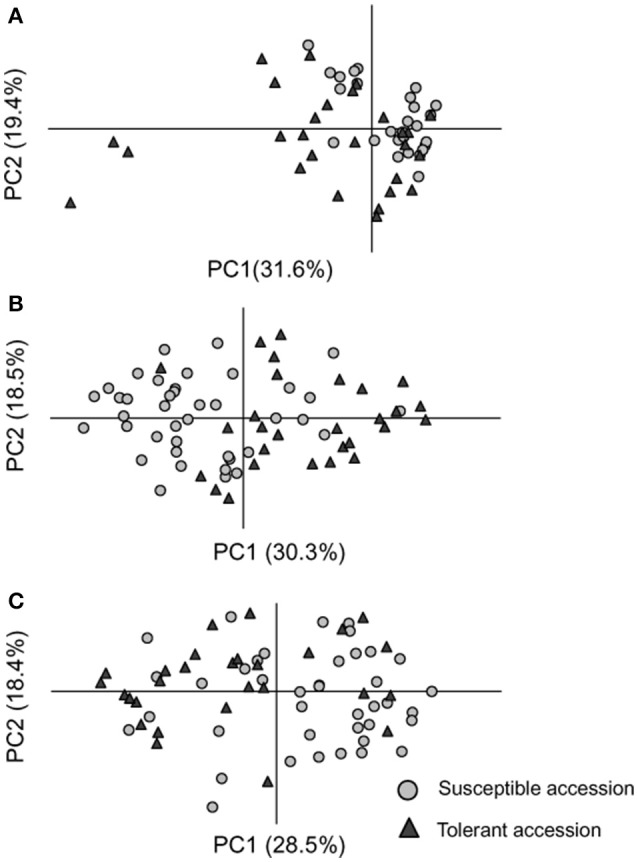
**Separations of susceptible (gray cycle) and tolerant (black triangle) accessions by the Principal Coordinate Analysis under the normal (CK) (A)**, stressed (OS) **(B)**, and recovery (RO) **(C)** conditions using differentially methylated epiloci.

Fourteen epiloci were determined as differentially methylated epiloci between tolerant and susceptible groups for each CK, OS, and RO treatments (**Tables 6**, S9). Generally, no apparent epigenetic difference between tolerant and susceptible accessions were found in the CK, OS, and RO conditions as suggested by the PCoA when total epiloci were used (Figure S4). However, tolerant and susceptible rice accessions were apparently separated by the PCoA in all three treatments by their differentially methylated epiloci (Figure 3). The differentially methylated epiloci in the three treatments rarely overlapped, suggesting that epigenetic responses in the CK, OS, and RO treatmentsmay be associated with different epiloci (Figure S5).

Sixteen epiloci were determined as tolerance-associated epiloci in the CK treatment, since rice accessions conferred different epigenotypes at these loci and exhibited different survival ratios (Table 5). Six of these were determined as differentially methylated epiloci (Table 6). Similarly, 13 and 17 tolerance-associated epiloci were detected in OS and RO treatments (Table 5), five and four among which were determined as differentially methylated epiloci (Table 6). The higher proportion of tolerance-associated epiloci indicated that differentially methylated epiloci had an increased probability to be associated with tolerance to osmotic stress than others. It is worth noting that the epigenotypes of lower methylation degrees on these differentially methylated epiloci in the OS treatments resulted in higher survival ratios, while epigenotypes of higher methylation degrees on most differentially methylated epiloci in RO represented higher survival ratios (Table 6). These results were consistent with the results of correlation analyses in which the survival ratio was negatively and positively correlated with the DNA methylation level in OS and ratio of re-methylation events from OS to RO.

**Table 5:** **Number of tolerance-associated epiloci and their proportions in differentially methylated epiloci and total epiloci in normal, osmotic-stressed, and recovery treatments**.

**Condition**	**Number in DME**	**Proportion in DME**	**Number in other loci**	**Proportion in other loci**	**Number in total loci**	**Proportion in total loci**
Normal	6	0.429	10	0.056	16	0.055
Osmotic-stressed	5	0.357	8	0.045	13	0.044
Recovery	4	0.286	13	0.047	17	0.058
Average	5	0.357	10.3	0.049	15.3	0.052

**Table 6:** **Epigenotypes on differentially methylated epiloci have significantly different survival ratios in normal (CK), stressed (OS), and recovery (RO) conditions**.

**Condition**	**Candidate epilocus**	**Epigenotype**	**Frequency**	**Survival ratio (%)**
CK	EL039	0/1	59/68	10.6 ± 1.5^*^
		1/1	9/68	20.1 ± 3.8^*^
	EL080	0/1	6/67	4.8 ± 1.2^*^
		1/1	57/67	12.5 ± 1.6^*^
	EL192	0/0	10/67	20.9 ± 4.6^*^
		0/1	56/57	10.4 ± 1.5^*^
	EL201	0/0	43/65	9.2 ± 1.4^*^
		0/1	21/65	15.4 ± 3.3^*^
	EL253	1/0	7/68	22.3 ± 4.7^*^
		1/1	60/68	10.6 ± 1.5^*^
	EL265	0/0	6/68	5.9 ± 2.1a
		0/1	46/68	10.27 ± 1.6a
		1/1	15/68	19.34 ± 3.8b
OS	EL114	0/0	7/56	4.7 ± 1.2a
		0/1	11/56	13.6 ± 4.0b
		1/0	16/56	6.2 ± 2.1a
		1/1	22/56	14.2 ± 2.3b
	EL216	0/0	27/63	7.1 ± 1.9^*^
		0/1	33/63	16.5 ± 2.3^*^
	EL224	1/0	31/67	6.6 ± 1.6^*^
		1/1	33/67	15.3 ± 2.3^*^
	EL225	0/0	33/65	8.2 ± 1.8^*^
		0/1	32/65	15.4 ± 2.3^*^
	EL268	1/0	27/66	9.5 ± 1.8^*^
		1/1	27/66	16.5 ± 2.7^*^
RO	EL051	0/0	37/67	15.7 ± 2.2^*^
		0/1	23/67	7.8 ± 1.9^*^
	EL057	0/0	41/67	14.9 ± 2.1^*^
		0/1	22/67	7.7 ± 2.1^*^
	EL084	0/0	32/65	10.9 ± 2.0ab
		0/1	18/65	7.2 ± 2.5a
		1/0	10/65	18.4 ± 4.3b
	EL105	0/0	6/68	6.7 ± 3.2a
		1/0	9/68	20.6 ± 3.8b
		1/1	53/68	10.9 ± 1.7ab

Values with ^“*”^ or different letters indicate significance (p < 0.05) by independent t-test or one-way ANOVA (SNK method).

### Expression of candidate genes of different epigenotypes at differentially methylated epiloci in CK and OS conditions

Seventy-four DNA fragments were annotated to 31 scored epiloci, which occupied 83.8% of total scored MSAP epiloci from this selective primer combination (E14-HM36) (Table S5). Two genes at both candidate epiloci EL265 and EL268 were selected to quantify their expressions via qPCR (Table S6) in at least five rice accessions subjected to both CK and OS treatments.

**Figure 4:**
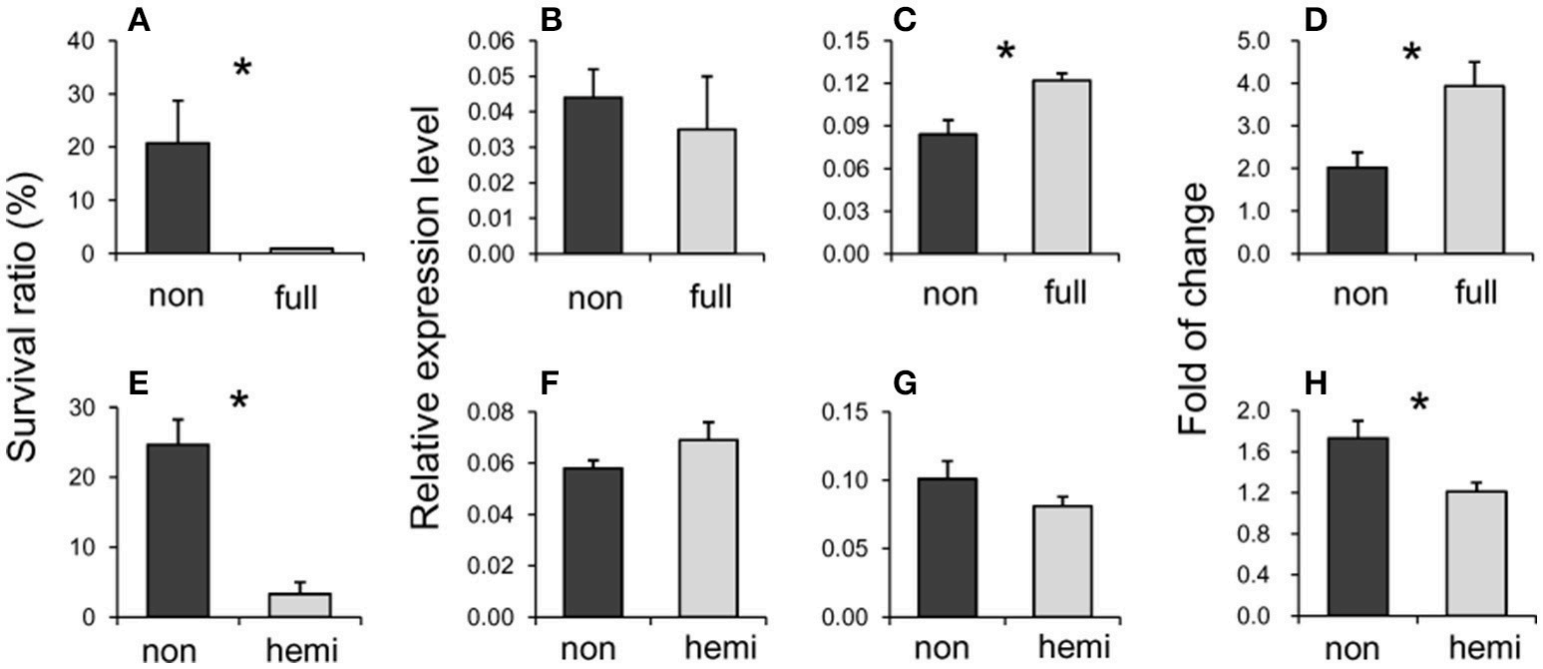
**Survival ratios (A,E)**, expression levels under the normal (CK) **(B,F)** and stressed (OS) **(C,G)** conditions, and fold changes from CK to OS **(D,H)** of two epigenotypes on EL265 (annotated as LOC_Os03g51020) **(A–D)** and EL 268 (annotated as LOC_Os07g16224) **(E–H)**. Asterisks indicate significant differences between tolerant and susceptible groups. Full indicates full-methylation, non indicates non-methylation, hemi indicates hemi-methylation.

EL265 was detected as DME in the CK condition. It located at −1412 bp from the translation start site of LOC_Os03g51020. This epilocus was mainly de-methylated in the susceptible group (37.5% de-methylated events and 17.5% re-methylated events), while the proportions of re-methylated (21.4%) and de-methylated (25.0%) events on this locus were similar in the tolerant group when osmotic stress was encountered. The selected seedlings of 1/1 (non-methylation) epigenotypes in the CK condition had significantly higher survival ratios compared to that of 0/1 (full-methylation) epigenotypes (Table S6, Figure 4A). Its expression was significantly altered in 6 out of 10 rice accessions (Table S6). Surprisingly, its expression level was not different to CK conditions between both epigenotypes (Figure 4B) although a differentially methylation was detected in CK. However, the expression was significantly higher in the susceptible epigenotype than in the tolerant epigenotype in OS (Figure 4C), as well as its fold changes from CK to OS (Figure 4D). Based on the Rice Functional Related gene Expression Network database, LOC_Os03g51020 co-expressed with three Snf2 family proteins (LOC_Os01g27040, LOC_Os08g08220, and LOC_Os07g31450). These genes play a vital role in regulating DNA methylation and pose profound impact on plant development.

EL268 was detected as DME in the OS condition and is located at the genebody of LOC_Os07g16224. The proportions of re-methylated (34.2%) and de-methylated (28.9%) events from CK to OS on this locus were similar to the proportions found for the susceptible group. However, this epilocus was mainly de-methylated in the tolerant group from CK to OS (64.3% de-methylated events and 21.4% re-methylated events). The selected seedlings of 1/1 (non-methylation) epigenotypes in the OS condition had a significantly higher survival ratio than those of 1/0 (hemi-methylation) epigenotypes (Table 6, Figure 4E) and expression was significantly altered in seven out of 11 rice accessions (Table S6). Expressions in CK (Figure 4F) and OS (Figure 4G) were similar between tolerant and susceptible epigenotypes of this epilocus, while its fold change from CK to OS was significantly higher in tolerant epigenotypes (Figure 4H). Additionally, the top 50 genes (Table S10) of co-expression analyzed based on rice FREND mainly referred to biological processes, such as “DNA repair” (GO:0006281, FDR = 0.021), “response to DNA damage stimulus” (GO:0006974, FDR = 0.021), and “response to endogenous stimulus”(GO:0009719, FDR = 0.026).

## Discussion

### Severe alterations in physiological traits and genome-wide DNA methylation as rice encounters and recovers from osmotic stress

Consistent with previous studies, the relative water content decreased while the total soluble protein, contents of H_2_O_2_, MDA, and total antioxidant capacity increased as rice encountered osmotic-stress (Huang et al., 2009; Du et al., 2010; Li et al., 2016). It is surprising that the H_2_O_2_ content, which has always been considered toxic for plants (You and Chan, 2015), was significantly higher in tolerant accessions for both normal and stress treatments. However, H_2_O_2_ also acts as a signaling molecule (Hossain et al., 2015; You and Chan, 2015; Saxena et al., 2016). The accumulation of H_2_O_2_ could enhance plant tolerance to drought stress by regulating stomatal closure and promoting antioxidant systems, ultimately resulting in higher survival ratios. The higher content of H_2_O_2_ was observed in the *dst* mutant (Huang et al., 2009) and the over-expression line of *OsARS5* (Li et al., 2016), both exhibited increased tolerance compared to the wild-type. Such a promotion of antioxidant systems was also observed in our study. Furthermore, higher contents of H_2_O_2_ in tolerant accessions could be a reflection of their better tolerances to ROS generated from normal life activities under dehydration.

Plants are known to alter their genome-wide DNA methylation in response to various abiotic stresses (Angers et al., 2010; Dowen et al., 2012). Accompanied by alterations of physiological traits, more than half of the individual-epilocus combinations of rice altered their methylation status in response to encountering or recovering from osmotic stress in this study. This is similar to results recorded for rice encountering drought stress (Wang et al., 2011; Zheng et al., 2013) or salt stress (Karan et al., 2012). Many previous studies reported a decrease of DNA methylation levels when rice encountered drought stress regardless of whether these were tolerant or susceptible genotypes (Wang et al., 2011; Zheng et al., 2013). However, another study reported strongly increased overall methylation levels when rice seedlings encountered drought stress (Gayacharan and Joel, 2013). In our study, observed from considerable number of rice genotypes, the general methylation level of rice seedlings was only slightly enhanced (below statistical significance) in responses to osmotic stress, while it strongly increased when rice recovered from osmotic stress. This result indicates that the recovery from the osmotic stress is not a simple reversible process of rice in responses to the stress.

### Differentially methylated epilocus generated from numerous genotypes of contrasting tolerance have a high probability to be associated with osmotic tolerance

The association of DNA methylation in response to a given stress leading to plant tolerance has been widely discussed (Kou et al., 2011; Karan et al., 2012; Wang et al., 2015), including for rice tolerances to drought stress (Wang et al., 2011; Gayacharan and Joel, 2013; Garg et al., 2015). However, studies using a limited number of rice genotypes exhibited inconsistent results. E.g., one study reported very similar general methylation levels between DK151 (tolerant genotype) and IR64 (susceptible genotype) (Wang et al., 2011), while other studies reported lower DNA methylation levels or hypo-methylation in drought-tolerant genotypes under both normal and stressed conditions (Gayacharan and Joel, 2013; Zheng et al., 2013; Wang W. et al., 2016). These inconsistent results are suggested to be due to genotype-specific manners of DNA methylation (Karan et al., 2012). The genotype-specific manner of DNA methylation also causes great variation of DNA methylation between two genotypes of contrasting tolerances (Wang et al., 2011; Garg et al., 2015). E.g., 64212 (N22/IR64) and 35723 (PK/IR64) differentially methylated regions (DMR) were identified between two pairs of different cultivars via whole genome bisulphite sequencing, which covered 57.9 and 38.5% protein coding genes annotated in the reference genome (Garg et al., 2015). Furthermore, large proportions of DMRs detected between two cultivars were reported not stress-relevant, as they were not in response to drought-stress (Wang W. et al., 2016). It is therefore difficult to determine whether these differences in DNA methylation between two genotypes are associated with their contrasting tolerances or merely with a different genetic background.

In this study, the similarity between a tolerant and a susceptible genotype was approximately 50% on average for all three conditions. Consequently, we could generate ~140 differentially methylated epiloci between two rice genotypes per treatment. However, only approximately 5% of total epiloci were determined to be associated with osmoticstress tolerance. This result indicates that most of these differentially methylated epiloci between two genotypes could rarely be associated with stress tolerance. On the contrary, the differentially methylated epiloci, generated from a considerable number of genotypes, could be associated with stress-tolerance with high probability (e.g., 35.7% in this study). It is worth noting that the DME in CK, OS, and RO conditions hardly overlapped, suggesting that different epigenetic mechanisms were involved in rice responses to osmotic stress as well as during recovery.

### Differentially methylated epiloci impact gene regulations and contribute to increased tolerance

Although, the general methylation level was similar between tolerant and susceptible groups under both normal and stressed conditions, significantly more de-methylation events were observed in the tolerant group during osmotic stress, leading to a lower DNA methylation level compared to the susceptible group. Accordingly, the survival ratio was negatively correlated with the methylation level weekly. Interestingly, rice accessions conferring epigenotypes of lower methylation degrees on these DME under the OS condition always represented higher survival ratios. This result strongly indicates that lower methylation levels on these DME under stressed-condition contribute to higher osmotic-tolerance. Unlike DME during stress, rice accessions with epigenotypes of high methylation degree at the DME during recovery, did not always also have higher survival ratios, although re-methylation events appeared more frequently in the tolerant group.

DNA methylation could potentially cause adaptive physiological and morphological responses to stress (Angers et al., 2010; Ou et al., 2012; Róis et al., 2013) *via* regulating gene expressions (Zhao and Han, 2009; Garg et al., 2015). However, the influence on gene expression caused by DNA methylation varied among different genotypes under abiotic stress (Karan et al., 2012). In this study, we successfully annotated 91 genes to 31 epiloci in one selective primer combination, most of which encoded retrotransposon proteins. Moreover, two epiloci (LOC_Os03g51020 and LOC_Os07g16224) were determined as DME and can be considered to be associated with osmotic stress tolerance. Although great variations of expressions on both genes were also observed among rice accessions, different epigenotypes of these two differentially methylated loci significantly impacted their gene regulations (fold changes) when rice encountered osmotic stress. LOC_Os03g51020 encodes an expressed Ser-Thr protein kinase-like protein and it co-expresses with several Snf2 family proteins based on RiceFREND. Snf2 family proteins play a role in regulations of DNA methylation (Hu et al., 2013). Differential methylation and regulation on this epilocus (gene) as rice is subjected to stress may result in genome-wide differentially methylated epiloci. Additionally, one of these Snf2 family protein-encoding genes (LOC_Os07g31450) has been revealed to influence rice crown root development (Wang Y. H. et al., 2016), which is associated with rice adapting to drought stress (Gao and Lynch, 2016). LOC_Os07g16224 encodes a rice Argonaute protein (*OsAGO16*). The results of Rice FREND suggest its co-expression with *OsTOP6B* (LOC_Os09g10770). Over-expression of *OsTOP6B* in *A. thaliana* has been reported to improve its stress-tolerance (Jain et al., 2006). In addition, *OsAGO16* can be assumed to be relevant for DNA damage repair in rice. These results suggest that rice accessions conferring the epigenotype of lower methylation degree could contribute to osmotic stress tolerance by regulating gene expressions. However, we did not find these two genes in the DMR-associated DEGs (differentially expressed between cultivars) in a previous study (Garg et al., 2015). This could be due to genotype-specific of DNA methylation or their similarity of expressions between tolerant and susceptible genotypes in the stressed treatment.

### Significance of epigenetic markers of DNA methylation in the evolution and breeding of rice drought-resistance

Epigenetic mechanisms play important roles in the adaptations of plants to various stresses and are significant in the evolution of emerging tolerance (Bräutigam et al., 2013; Trick, 2015). In a previous study, we detected lower methylation levels on differentially methylated epiloci in the upland rice ecotype (Xia et al., 2016). The upland rice confers higher drought-resistance compared to lowland rice. Based on the findings of this study, we conclude that lower DNA methylation levels in stressed condition contribute to higher drought-resistance in upland rice. The differentially methylated epiloci found in this study should be good epigenetic markers to decipher the evolution of drought-resistance in rice, which could not fully explained by genetic differentiation (Xia et al., 2014).

The application of epigenetic mechanisms in crop breeding for stress-tolerances has received particular attention during recent years (Peng and Zhang, 2009; Shi and Lai, 2015; Bilichak and Kovalchuk, 2016). Plants may enhance their tolerances to biotic/abiotic stresses via transgenerational epigenetic mechanisms when ancestors are exposed to the specific stress in their environment (Holeski et al., 2012; Trick, 2015). E.g., DNA methylation mediated tolerance enhancement has been observed in rice as a response to nitrogen-deficiency (Kou et al., 2011) and heavy metal exposure (Ou et al., 2012). In a previous study, a susceptible rice genotype domesticated in artificial drought stress for successive generations, maintained 58.3% de-methylation events inherited from its stress-treated parents (Zheng et al., 2013), potentially altering its drought-resistance (Zheng et al., 2017). Our findings of low methylation degree associated drought-tolerance can well explain these findings. Using epigenetic markers in rice breeding of drought-resistance is a highly feasible method. However, more stable and informative epigenetic markers could only generated from numerous genotypes via high-throughput techniques. They are required for future breeding.

## Authors contributions

HX designed this experiment, analyzed the data, and drafted this manuscript. WH carried out most of this experiment and participated in the data analyses and manuscript drafting. JX, SY, TT, JL, and JW performed some parts of the experiment and were involved in manuscript drafting. LL was involved in the design of this experiment and manuscript drafting. All the authors have revised this manuscript critically before the submission and agreed with all aspects of the work.

## Funding

This research was supported by Shanghai Agricultural Key Science and Technique Project (Shanghai Key Agricultural Science (2016) 6-1-7), Project of Shanghai Talent Youth of Agriculture, 2015 (Grant No. Shanghai Youth Agricultural Science (2015) 1–5), Project of Subject Construction, Shanghai Academy of Agricultural Sciences, 2015, 2016 (Grant No. SAAS-2015(07), SAAS-2016(07)), and National High-Tech Research and Development Program of China (863 Plan) (Grant No. 2014AA10A603, 2014AA10A604), and the National Program for Basic Research of China (2012CB114305). The funders had no role in study design, data collection and analysis, decision to publish, or preparation of the manuscript.

### Conflict of interest statement

The authors declare that the research was conducted in the absence of any commercial or financial relationships that could be construed as a potential conflict of interest.

## Supplementary material

Figure S1 **Procedures of rice cultivation and experimental treatments**.

Figure S2 **The frequency of counts to a DNA fragment among total fragments sequenced from the PCR product**.

Figure S3 **The frequency of survival ratios among the 68 rice accessions**.

Figure S4 **Separations of susceptible (gray cycle) and tolerant (black triangle) accessions by the Principal Coordinate Analysis under the normal (CK) (A)**, stressed (OS) **(B)**, and recovery (RO) conditions using total epiloci.

Figure S5 **The number of differentially methylated epiloci detected under the normal (CK), stressed (OS), and recovery (RO) conditions and their overlaps**.

Online Supplementary Data Sheet 1 **Original MSAP scoring matrices of rice accessions in CK, OS, and RO conditions**.

Table S1 **Composition of the nutrient solution**.

Table S2 **Information of selective primer combinations involved in this study**.

Table S3 **Brief procedures of measurements for physiological traits**.

Table S4 **Alterations of MSAP epigenotypes from CK to RO in different groups**.

Table S5 **DNA fragments of scored MSAP epiloci identified by direct sequencing and their basic information**.

Table S6 **Expressions of genes at differentially methylated under CK and OS conditions**.

Table S7 **Original data of the phenotypes for rice accessions**.

Table S8 **Correlations between parameters of DNA methylation and physiological traits**.

Table S9 **Epigenotypes of some differentially methylated epiloci have no influence on the survival ratio in normal, stressed, and recovery conditions**.

Table S10 **Top 50 co-expressing genes with two candidate genes in our study by RiceFREND**.
