# Supplementary figures and images for: Differentially Methylated Epiloci Generated from Numerous Genotypes of Contrasting Tolerances Are Associated with Osmotic-Tolerance in Rice Seedlings
Source: Front Plant Sci. 2017 Jan 19;8:11. doi: 10.3389/fpls.2017.00011 (PMC5243842; doi:10.3389/fpls.2017.00011)

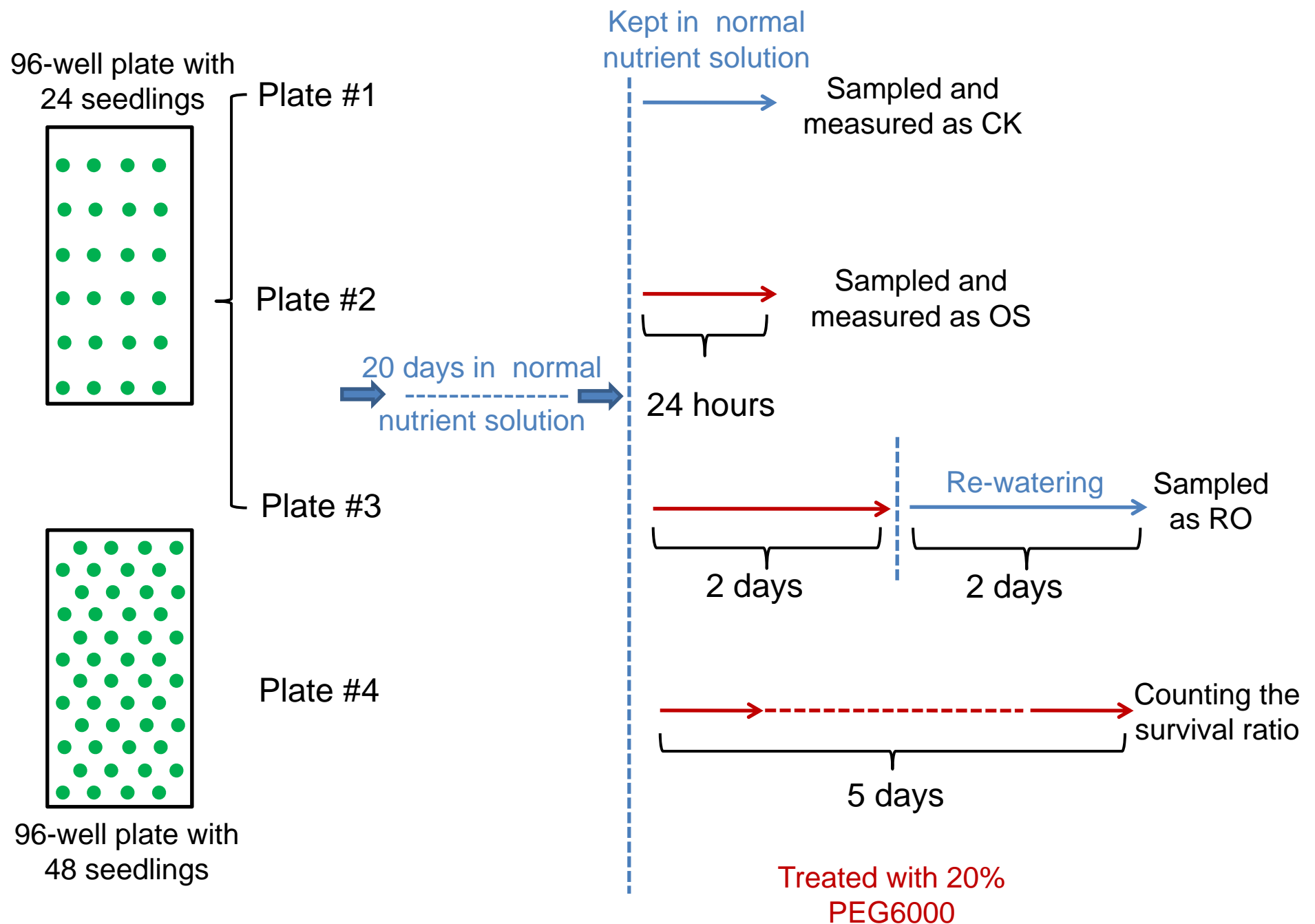

Fig. S1

Supplement: Figure S1 — Procedures of rice cultivation and experimental treatments. [file Image1.PDF]

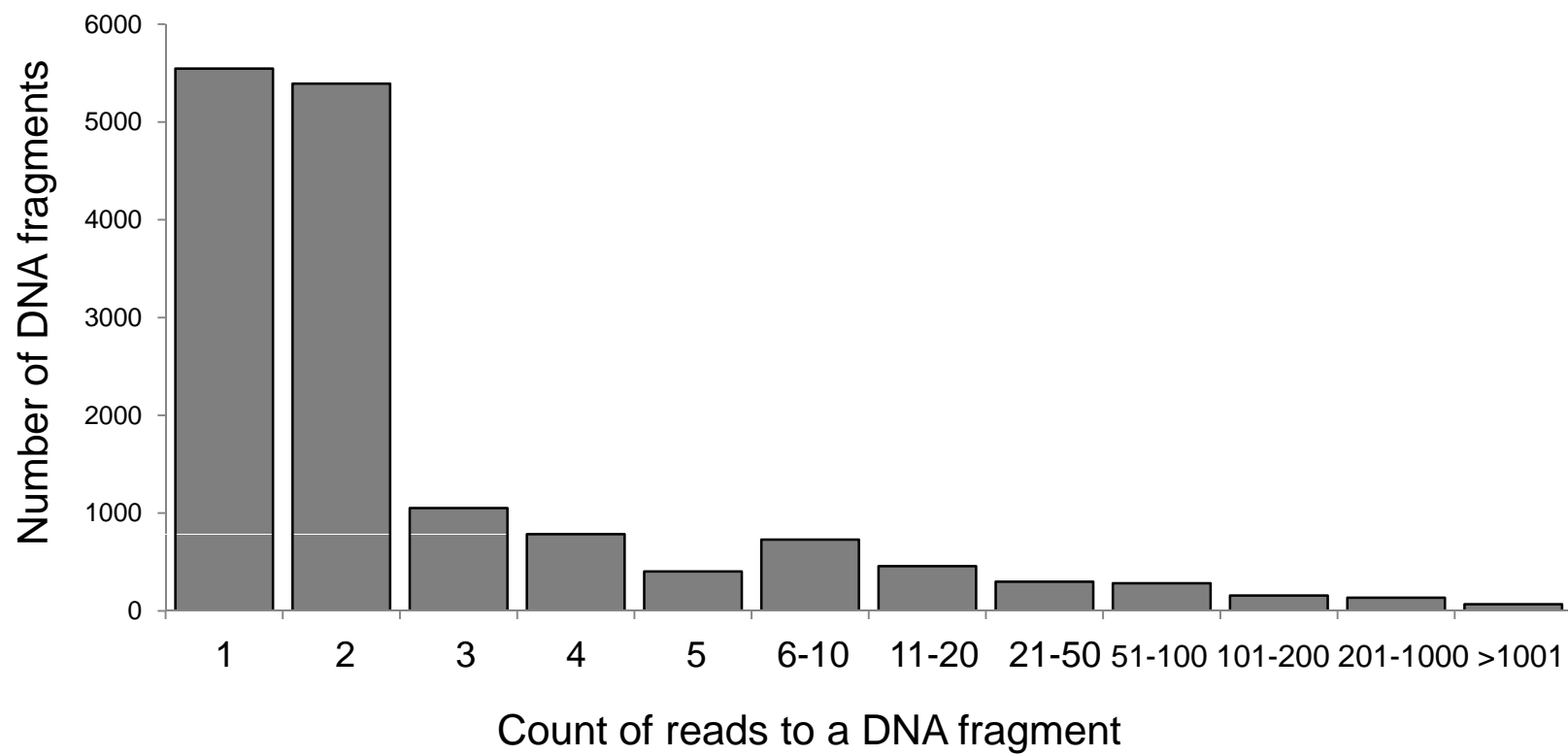

Fig. S2

Supplement: Figure S2 — The frequency of counts to a DNA fragment among total fragments sequenced from the PCR product. [file Image2.PDF]

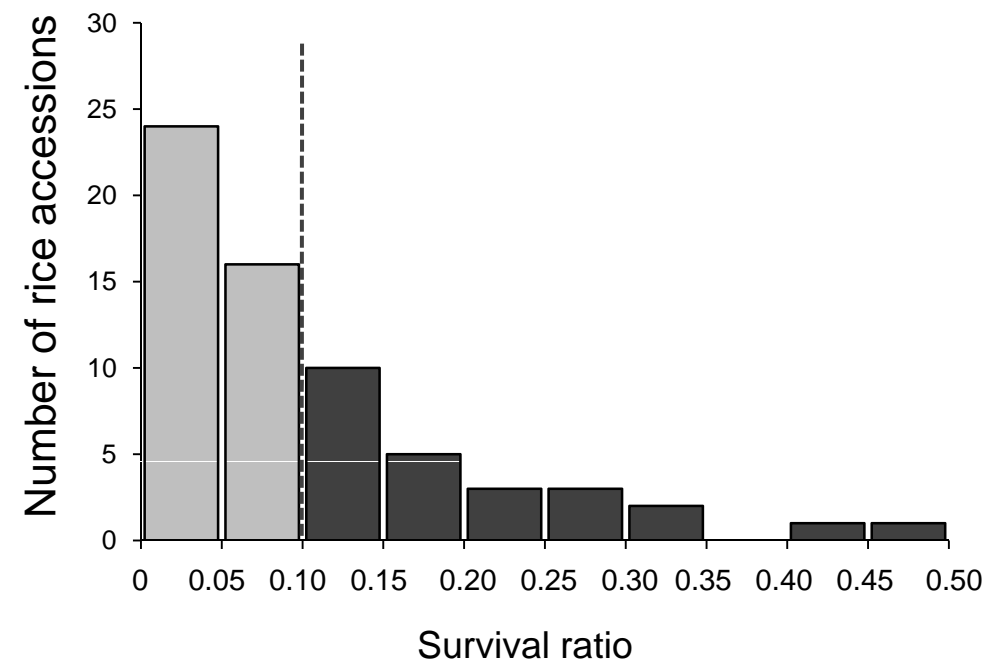

Fig. S3

Supplement: Figure S3 — The frequency of survival ratios among the 68 rice accessions. [file Image3.PDF]

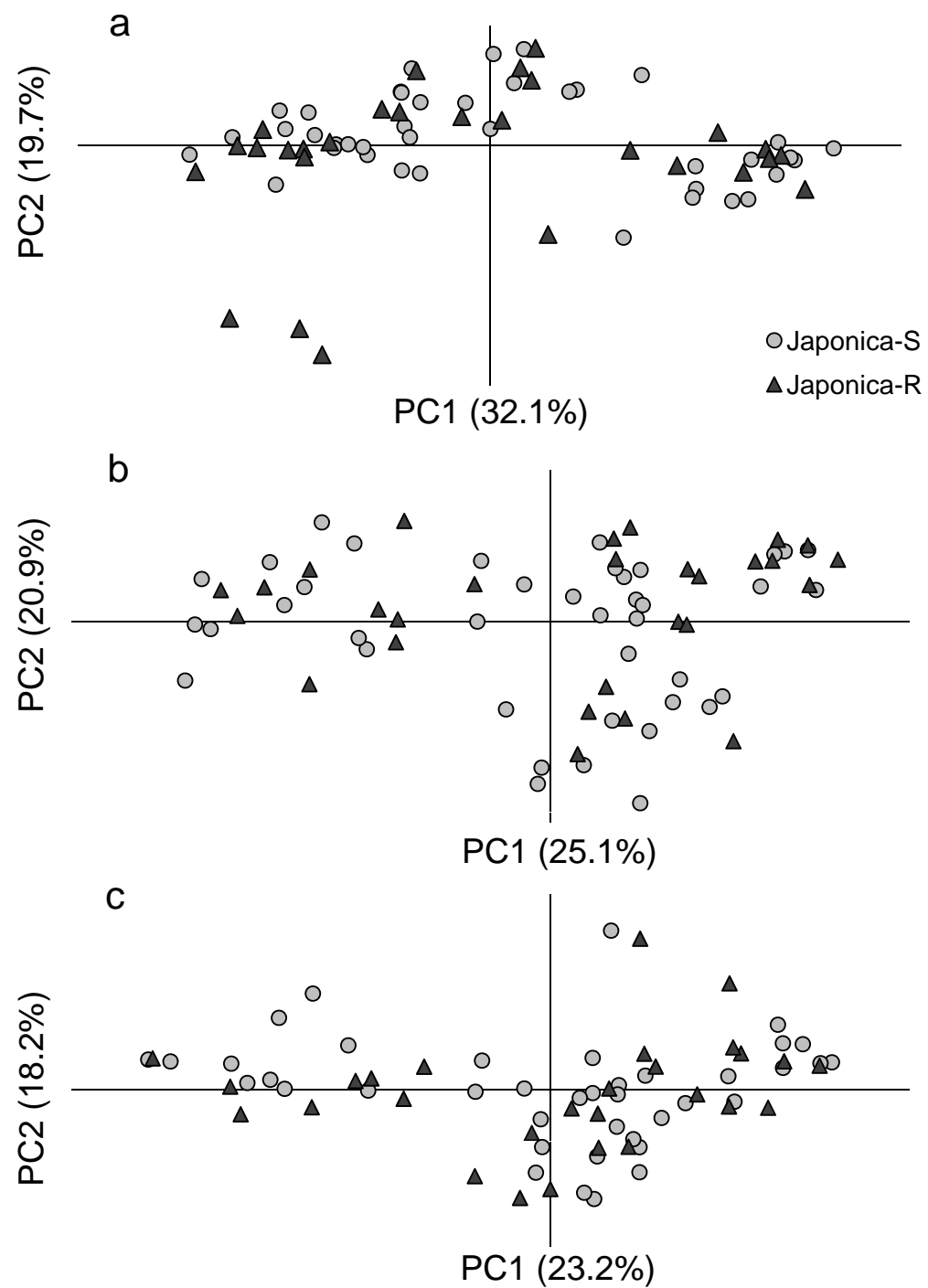

Fig. S4

Supplement: Figure S4 — Separations of susceptible (gray cycle) and tolerant (black triangle) accessions by the Principal Coordinate Analysis under the normal (CK) (A), stressed (OS) (B), and recovery (RO) conditions using total epiloci. [file Image4.PDF]

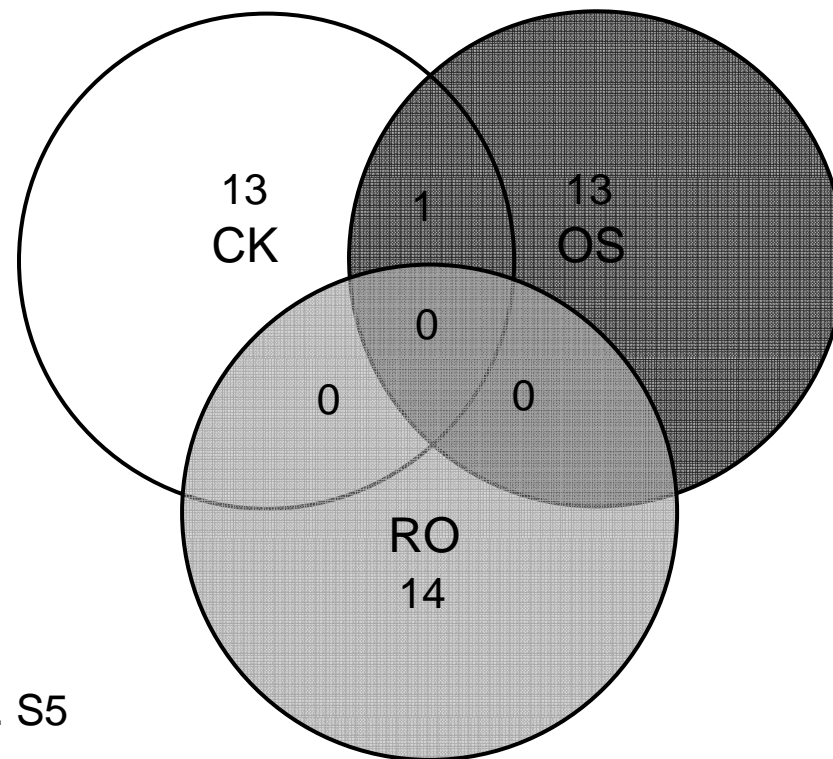

Fig. S5

Supplement: Figure S5 — The number of differentially methylated epiloci detected under the normal (CK), stressed (OS), and recovery (RO) conditions and their overlaps. [file Image5.PDF]
