# Supplementary material for: Differentially Methylated Epiloci Generated from Numerous Genotypes of Contrasting Tolerances Are Associated with Osmotic-Tolerance in Rice Seedlings
Source: Front Plant Sci. 2017 Jan 19;8:11. doi: 10.3389/fpls.2017.00011 (PMC5243842; doi:10.3389/fpls.2017.00011)
Supplement: Table S1 — Composition of the nutrient solution. [file Table1.DOCX]

Table S1 The composition of the normal nutrient solution.

| Solution | Chemical or component | Content in 1L solution |
| --- | --- | --- |
| Solution part 1 | CaCl_2_ | 88.6 g |
|  | NH_4_NO_3_ | 97.3 g |
| Solution part 2 | NaH_2_PO_4_·2H_2_O | 40.3 g |
|  | K_2_SO_4_ | 71.4 g |
| Solution part 3 | MgSO_4_·7H_2_O | 324 g |
| Solution part 4 | MnCl2·4H_2_O | 1.5 g |
|  | (NH4)_6_Mo_7_O_24_·4H_2_O | 0.074 g |
|  | H_3_BO_4_ | 0.934 g |
|  | ZnSO_4_·7H_2_O | 0.035 g |
|  | CuSO_4_·5H_2_O | 0.031 g |
|  | FeCl_3_·3H_2_O | 7.7 g |
|  | Na_3_C_6_H_5_O_7_·2H_2_O | 11.9 g |
| Final nutrient solution | Solution part 1 | 1.25 ml |
|  | Solution part 2 | 1.25 ml |
|  | Solution part 3 | 1.25 ml |
|  | Solution part 4 | 1.25 ml |
|  | 12.5 mol HCl | 0.1ml (to ~PH6.0) |
