# Supplementary material for: Differentially Methylated Epiloci Generated from Numerous Genotypes of Contrasting Tolerances Are Associated with Osmotic-Tolerance in Rice Seedlings
Source: Front Plant Sci. 2017 Jan 19;8:11. doi: 10.3389/fpls.2017.00011 (PMC5243842; doi:10.3389/fpls.2017.00011)
Supplement: Table S3 — Brief procedures of measurements for physiological traits. [file Table3.DOCX]

**Table S3 Procedures for measurements of physiological traits**

**Procedure for MDA measurement**

**1. Sample preparation**

0.1g rice leaf tissue was smashed by the grinder and mixed with 900ul 10×diluted regent-5. The mixture was then centrifuged by 4000rpm for ten minutes. The supernatant was kept at ice-bath for further use.

**2. Regent preparation**

The working regent was mixed with regent-1, regent-2, and regent-3 with a ratio of 0.1:3:1.

**3. Measurement**

The sample was composed by 50ul supernatant from the leaf tissue and 1000ul working regent. The standard was composed by 50ul MDA standard (10nmol/ml) and 1000ul working regent. The blank control was composed by 50ul absolute ethyl alcohol and 1000ul working regent. The mixture was put into 95℃ water-bath for twenty minutes and then measured the absorbance at 530 nm.

**4. Calculation**

The content of MDA in sample = (OD_sample_ - OD_blank_)/(OD_standard_- OD_blank_)*standard

**Procedure for total soluble protein measurement**

**1. Sample preparation**

0.1g rice leaf tissue was smashed by the grinder and mixed with 900ul 5% saline. The mixture was then centrifuged by 2500rpm for ten minutes. The supernatant was kept at ice-bath for further use.

**2. Regent preparation**

The regent-1 (Coomassie brilliant blue) was diluted to 5 times.

**3. Measurement**

The sample was composed by 50ul supernatant from the leaf tissue and 3000ul diluted Coomassie brilliant blue. The standard was composed by 50ul protein standard (0.563g/L) and 3000ul Coomassie brilliant blue. The blank control was composed by 50ul saline and 3000ul Coomassie brilliant blue. The mixture was kept at room condition for ten minutes and then measured the absorbance at 595 nm.

**4. Calculation**

The content of MDA in sample = (OD_sample_ - OD_blank_)/(OD_standard_- OD_blank_)*standard

**Procedure for H_2_O_2_ measurement**

**1. Sample preparation**

0.1g rice leaf tissue was smashed by the grinder and mixed with 900ul 5% saline. The mixture was then centrifuged by 10000rpm for ten minutes. The supernatant was kept at ice-bath for further use.

**2. Measurement**

The sample was composed by 1000ul regent-1, 100ul supernatant from the leaf tissue, and 1000ul regent-2. The standard was composed by 1000ul regent-1, 100ul H_2_O_2_ standard (16.3mmol/L), and 1000ul regent-2. The blank control was composed by 1000ul regent-1, 100ul saline, and 1000ul regent-2. The mixture was kept at room condition for ten minutes and then measured the absorbance at 405 nm.

**3. Calculation**

The content of MDA in sample = (OD_sample_ - OD_blank_)/(OD_standard_- OD_blank_)*standard

**Procedure for measurement of total anti-oxidant capacity**

**1. Sample preparation**

0.1g rice leaf tissue was smashed by the grinder and mixed with 900ul 5% saline. The mixture was then centrifuged by 3500rpm for ten minutes. The supernatant was kept at ice-bath for further use.

**2. Measurement**

The sample was composed by 1000ul regent-1, 100ul supernatant from the leaf tissue, and 2000ul regent-2, and 500ul regent-3. The blank control was composed by 1000ul regent-1, and 2000ul regent-2, and 500ul regent-3. The mixture was kept in 37℃ water-bath for 30 minutes. The sample mixture was then added with 200ul regent-4 and 200ul regent-5 while the blank control was then added with 200ul regent-4, 100ul supernatant from the leaf tissue, and 200ul regent-5. The mixture was kept at room condition for ten minutes and then measured the absorbance at 520 nm.

**4. Calculation**

The content of MDA in sample = (OD_sample_ - OD_blank_)/0.3*(total reaction volume/sample volume)/sample concentration
