# Supplementary material for: Differentially Methylated Epiloci Generated from Numerous Genotypes of Contrasting Tolerances Are Associated with Osmotic-Tolerance in Rice Seedlings
Source: Front Plant Sci. 2017 Jan 19;8:11. doi: 10.3389/fpls.2017.00011 (PMC5243842; doi:10.3389/fpls.2017.00011)
Supplement: Table S4 — Alterations of MSAP epigenotypes from CK to RO in different groups. [file Table4.DOCX]

**Table S4.** Alterations of MSAP epigenotypes from CK to RO in different groups.

| Material group | Alteration pattern | | | | |
| --- | --- | --- | --- | --- | --- |
|  | A-B-B | A-B-C | A-B-A | A-A-A | A-A-B |
| Susceptible | 17.5±0.8 | 18.0±0.5 | 15.4±0.7 | 23.4±0.6 | 25.6±0.8 |
| Tolerant | 17.7±1.0 | 19.5±0.9 | 15.4±0.8 | 23.5±1.2 | 23.8±1.0 |
| Overall | 17.5±0.6 | 18.6±0.5 | 15.4±0.5 | 23.4±0.6 | 25.1±0.6 |
